# Mammal parasites in arid Australia

**DOI:** 10.1016/j.ijppaw.2020.02.003

**Published:** 2020-03-25

**Authors:** Ian Beveridge

**Affiliations:** a Faculty of Veterinary and Agricultural Sciences, University of Melbourne, Victoria, Australia; b South Australian Museum, Adelaide, South Australia, Australia

**Keywords:** Helminths, Australia, Macropodids, Vombatids, Rodents, Arid zone

## Abstract

The helminth and arthropod parasite communities occurring in macropodid, vombatid and notoryctid marsupials as well as in rodents in the arid zone of Australia are compared with those found in related host species in adjacent semi-arid and humid zones and are also related, where possible, to the changes in the mammalian fauna of central Australia over a geological time scale. Across the marsupials and rodents for which parasitological data are available, there is an obvious contrast in the helminth communities between inhabitants of high rainfall areas and those inhabiting semi-arid and arid zones in terms of parasite genera present in the marsupials. The differences between the inhabitants of semi-arid and arid environment communities are less overt and are observable only the parasite species level in the case of the kangaroos and wallabies. In the case of the rodents, there appears to be a significant lack of diversity in helminth faunas associated with the transition to an arid environment. Differences in the arthropod parasite communities between climatic regions are more marked than is the case with the helminths. The general lack of life cycle studies of these parasites provides an impediment to identifying the means by which they have adapted to the increasing aridity in central Australia over geological time, but appears to offer opportunities for future study.

## Highlights

• Not really applicable to review articles.

## Graphical abstract

*Petrogale xanthopus*, the yellow-footed rock wallaby: an inhabitant of arid inland areas of eastern Australia.

**Figure:**
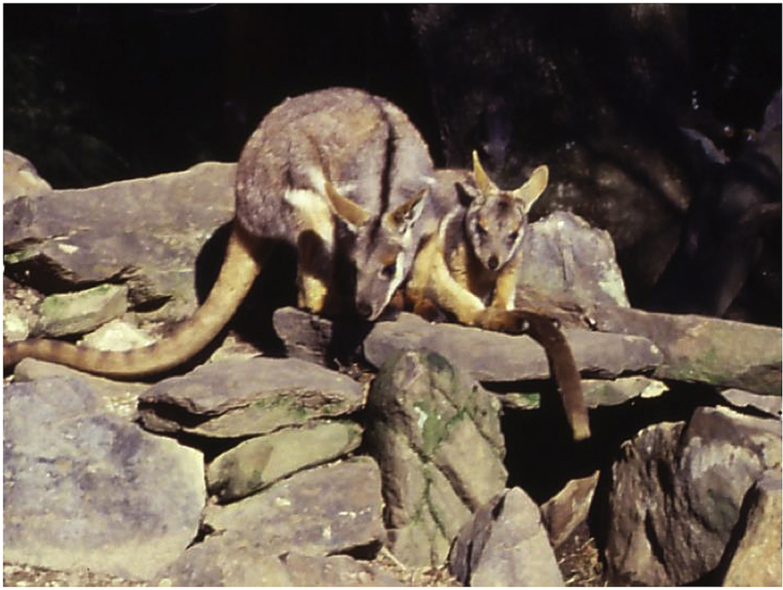

## 1 Introduction

Some 70% of the Australian landmass is classified as arid or semiarid (Barker and Greenslade, 1982), based on the evaporation/precipitation index of Prescott (1946), with the arid zone itself in the central region of the continent covering areas receiving less than about 250 mm of rainfall a year (Barlow, 1981). As a result, Australia has been described and the driest continent on earth. Yet, within this arid region, a substantial number of mammals, principally marsupials and rodents, survive and harbour parasites which successfully manage to complete their life cycles. As an example, the most prominent kangaroo species found in the arid zone, the red kangaroo, *Osphranter rufus*, is host to 18 genera and 31 species of helminths (Spratt and Beveridge, 2016) with average gastric nematode burdens of 58,000 (Arundel et al., 1979).

The adaptations which potentially underlie the success of these parasites are poorly understood, but are explored here insofar as the available evidence allows.

As background, it is necessary to define the geographical zone (the central Australian arid zone) under consideration, the mammals that inhabit this zone and then the parasites that appear to survive within it. The latter is approached by comparing the parasites of specific mammalian inhabitants of the arid zone with those of related species in adjacent semi-arid zones and the humid zones, with the humid zones being essentially coastal in their distribution.

## 2 Climatic zones in Australia

**Fig. 1:**
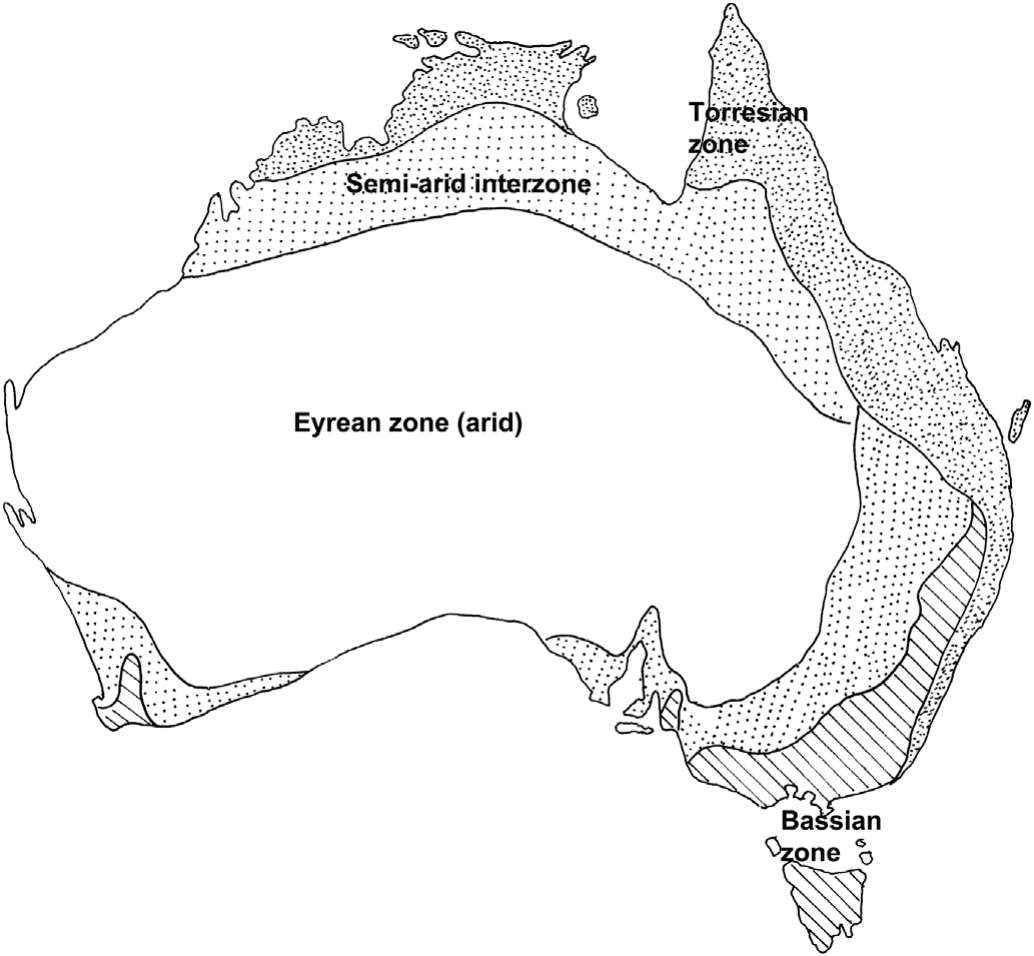
The Australian continent showing the major climatic zones defined by temperature, average rainfall and seasonality of rainfall (modified from Nix, 1982).

The Australian continent is divisible into a number of zones based either on biotic regions (Burbidge, 1960) or on a series of climatic parameters including temperature, rainfall and the seasonality of the rainfall (Nix, 1982). Whichever system is used, the overall results are similar. The central arid zone (the Eremaean or Eyrean zone) occupies most of the interior of the continent (Fig. 1). The climatic model used by Nix (1982) does not provide exactly the same as the definition of the arid zone used by Barlow (1981) based on the 250 mm isohyet, with the arid zone defined by Nix (1982) corresponding more closely to the 300 mm isohyet. However, this is a relatively minor distinction. The climatic zones have varied substantially over geological time. During the period up until about 5 mya (million years ago), central Australia was covered with rain forest and enjoyed summer rainfall and high humidities (Bowler, 1982). Subsequently, there was a shift towards winter rainfall as the continent migrated further north with increasing periods of aridity in central Australia and the replacement of forests by grasslands and shrublands. During the Pliocene (3–5 mya) there was seasonal winter aridity and then, during the Pleistocene (1–3 mya), there were major oscillations in the aridity of central Australia associated with the ice ages, the maximum aridity being reached 20,000 years ago (Bowler, 1982). It is against this background of continual climatic change and the shift from rain forest to desert in the centre of Australia that the mammal fauna of the region, and consequently their parasite fauna, has evolved.

## 3 The mammals of arid Australia

The original mammalian inhabitants of central Australia appear to have been rain-forest dwelling marsupials including macropodids (kangaroos and wallabies) and vombatoids (wombats and their relatives) (Hope, 1982). These were subsequently replaced, with increasing aridity, by grazing macropodids (kangaroos) and other groups of marsupials (wombats, dasyurids) better adapted to first of all the increase in grasslands and shrublands as the rain forests declined and then to the increasingly arid conditions in central Australia.

The rodent colonization of Australia from Papua New Guinea began about 5 mya (Rowe et al., 2008) more or less at the time when central forests were declining and the centre was becoming more arid. While several genera of rodents (*Melomys*, *Uromys*) remain as rainforest inhabitants in eastern Australia, other genera (*Notomys*, *Pseudomys*) have apparently adapted to and diversified in the arid conditions of central Australia (Baverstock, 1982).

The current mammalian fauna of central Australia (excluding bats) was documented by Baverstock (1982). Among the rodents, he considered that 14 of the 52 known Australian species were restricted to central Australia, while of the marsupials, potentially 12 of the 41 species of dasyurids, five of the 42 species of macropodids (kangaroos and wallabies), two of the nine bandicoot species (Peramelidae), two species of bilbies (Thalacomyidae) and the marsupial mole (*Notoryctes*) were restricted to arid Australia. No possums, pygmy possums, gliders (Phalangeridae, Pseudocheiridae, Burramyidae) or koalas (Phascolarctidae) occur in this region today. Baverstock (1982) excluded wombats, even though the southern hairy nosed wombat (*Lasiorhinus latifrons*) is restricted to arid and semi-arid habitats (van Dyck and Strahan, 2008). Some of the species Baverstock (1982) included are currently considered to be extinct (the pig-footed bandicoot, *Chaeropus ecaudatus*; the desert rat-kangaroo, *Caloprymnus campestris*) while recent taxonomic changes, particularly to the rock wallabies (*Petrogale* spp.), slightly increase the number of species and races restricted to central Australia (*P. purpureicollis*, the MacDonnell Ranges race of *P. lateralis*) (Eldridge et al., 2001). In addition, there are now considered to be two species of marsupial mole (Jackson and Groves, 2015). The data presented by Baverstock (1982) have been updated in (Table 1) allowing for taxonomic changes in host nomenclature since then (Jackson and Groves, 2015). However, the mammalian fauna when compared with the higher rainfall areas of the coastal regions remains somewhat depauperate.

**Table 1:** Mammals (marsupial and rodents) found primarily in the Australian arid zone^a^.

Marsupialia	
Dasyuridae	
*Dasycercus cristicaudata*	mulgara
*Dasykaluta rosamondae*	little red kaluta
*Dasyuroides byrnei*	kowari
*Pseudantechinus macdonellensis*	fat-tailed pseudantechinus
*Pseudantechinus woolleyi*	Woolley's pseudantechinus
*Phascogale calura*	red-tailed phascogale
*Planigale gilesi*	Giles' planigale
*Planigale tenuirostris*	narrow-nosed planigale
*Ningaui ridei*	wongai ningaui
*Ningaui timealeyi*	Pilbara ningaui
*Ningaui yvonnae*	southern ningaui
*Antechinomys laniger*	kultarr
*Sminthopsis crassicaudata*	fat-tailed dunnart
*Sminthopsis dolicura*	little long-tailed dunnart
*Sminthopsis hirtipes*	hairy-footed dunnart
*Sminthopsis longicaudata*	long-tailed dunnart
*Sminthopsis macroura*	stripe-faced dunnart
*Sminthopsis ooldea*	Ooldea dunnart
*Sminthopsis psammophila*	sandhill dunnart
*Sminthopsis youngsoni*	lesser hairy-footed dunnart
Myrmecobiidae	
*Myrmecobius fasciatus*	numbat
Peramelidae	
*Isoodon auratus*	golden bandicoot
*Perameles bougainville*	western barred bandicoot
*Macrotis lagotis*	bilby
Vombatidae	
*Lasiorhinus latifrons*	southern hairy-nosed wombat
Potoroidae	
*Bettongia lesueur*	burrowing bettong
*Bettongia penicillata*	brush-tailed bettong
Macropodidae	
*Lagorchestes hirsutus*	rufous hare-wallaby
*Osphranter robustus erubescens*	euro
*Osphranter rufus*	red kangaroo
*Petrogale burbidgei*	monjon
*Petrogale lateralis* Macdonell Ranges race	black-footed rock-wallaby
*Petrogale purpureicollis*	purple necked rock-wallaby
*Petrogale rothschildi*	Rothschild's rock-wallaby
*Petrogale xanthopus*	yellow-footed rock-wallaby
Family Notoryctidae	
*Notoryctes typhlops*	northern marsupial mole
*Notoryctes caurinus*	southern marsupial mole
**Eutheria**	
Family Muridae	
*Leggadina forresti*	Forrest's mouse
*Leporillus conditor*	greater stick-nest rat
*Notomys alexis*	spinifex hopping-mouse
*Notomys cervinus*	fawn hopping-mouse
*Notomys fuscus*	dusky hopping-mouse
*Pseudomys bolami*	Bolam's hopping-mouse
*Pseudomys chapmani*	western pebble-mound mouse
*Pseudomys desertor*	desert mouse
*Pseudomys fieldi*	Shark Bay mouse
*Pseudomys hermannsburgensis*	sandy inland mouse
*Pseudomys johnsoni*	central pebble-mound mouse
*Zyzomys pedunculatus*	central rock-rat
*Zyzomys woodwardi*	Kimberley rock-rat
*Rattus villosissimus*	long haired rat

a Based primarily on distribution data in van Dyck and Strahan (2008).

## 4 Helminths of marsupials and rodents

The extent of knowledge of the helminths of Australasian marsupials and monotremes was reviewed by Spratt and Beveridge (2016) who pointed out that there were no records of parasites from 26% of the known species of marsupials and this included a substantial number of species from arid Australia, particularly the dasyurids. Documentation of the parasites of Australian rodents has been somewhat neglected (Smales, 1997, 1998), particularly those of inland regions. Consequently, a study of the adaptations of parasites to the Australian arid zone is limited significantly by a lack of basic survey data. This issue is not limited to parasites, but is common to most free-living invertebrates in Australia's arid zone (Barker and Greenslade, 1982).

## 5 The kangaroos and wallabies (Macropodidae)

**Fig. 2:**
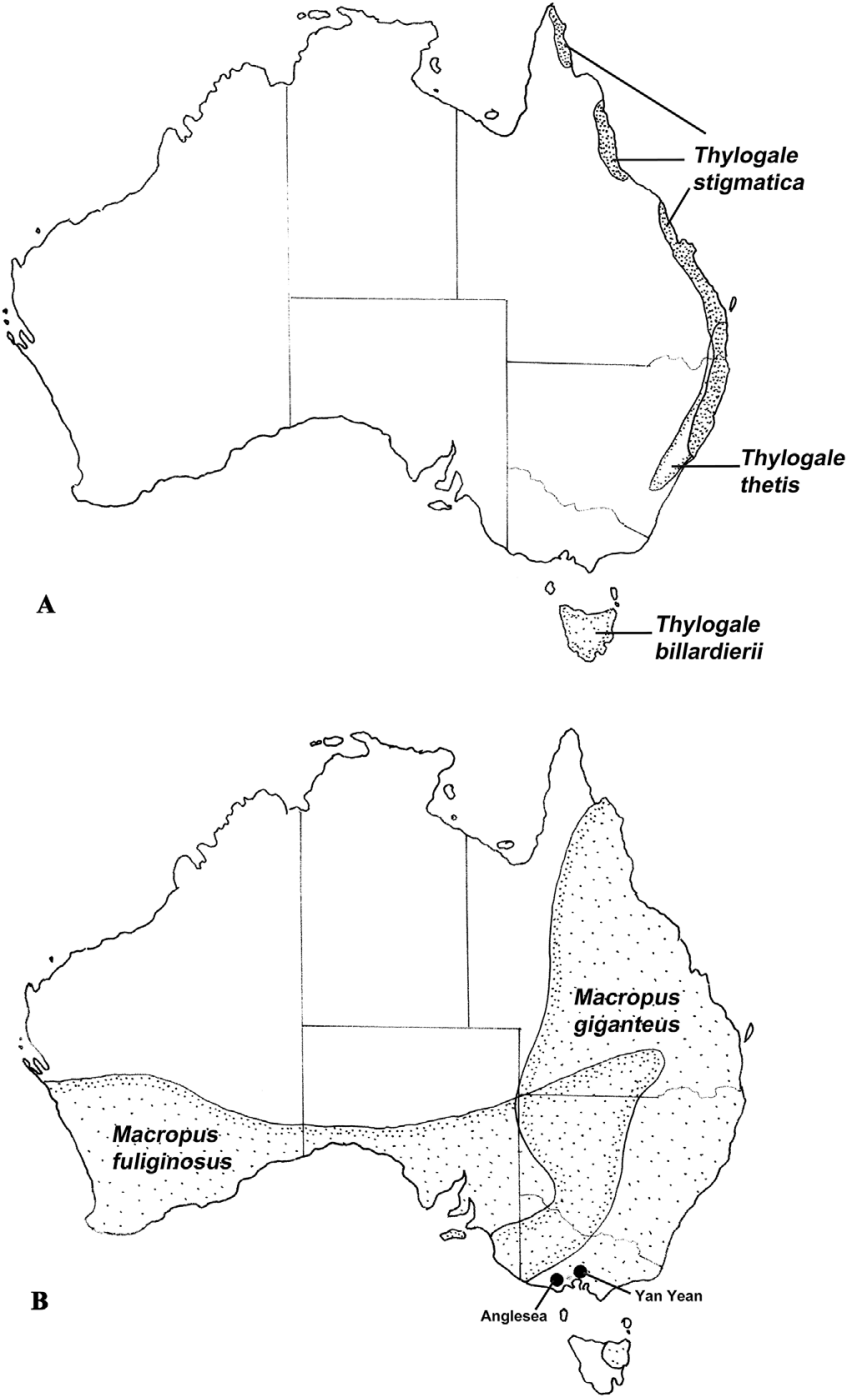
Geographical distributions of the rain-forest adapted pademelons (*Thylogale* spp.: Macropodidae) (A) and the grey kangaroos (*Macropus fuliginosus* and *M. giganteus* (Macropodidae) (B), with ranges of the latter two species extending into the semi-arid and arid ranges of the continent. Named localities are sites at which epidemiological studies of the parasites of these kangaroo species have been undertaken.

**Fig. 3:**
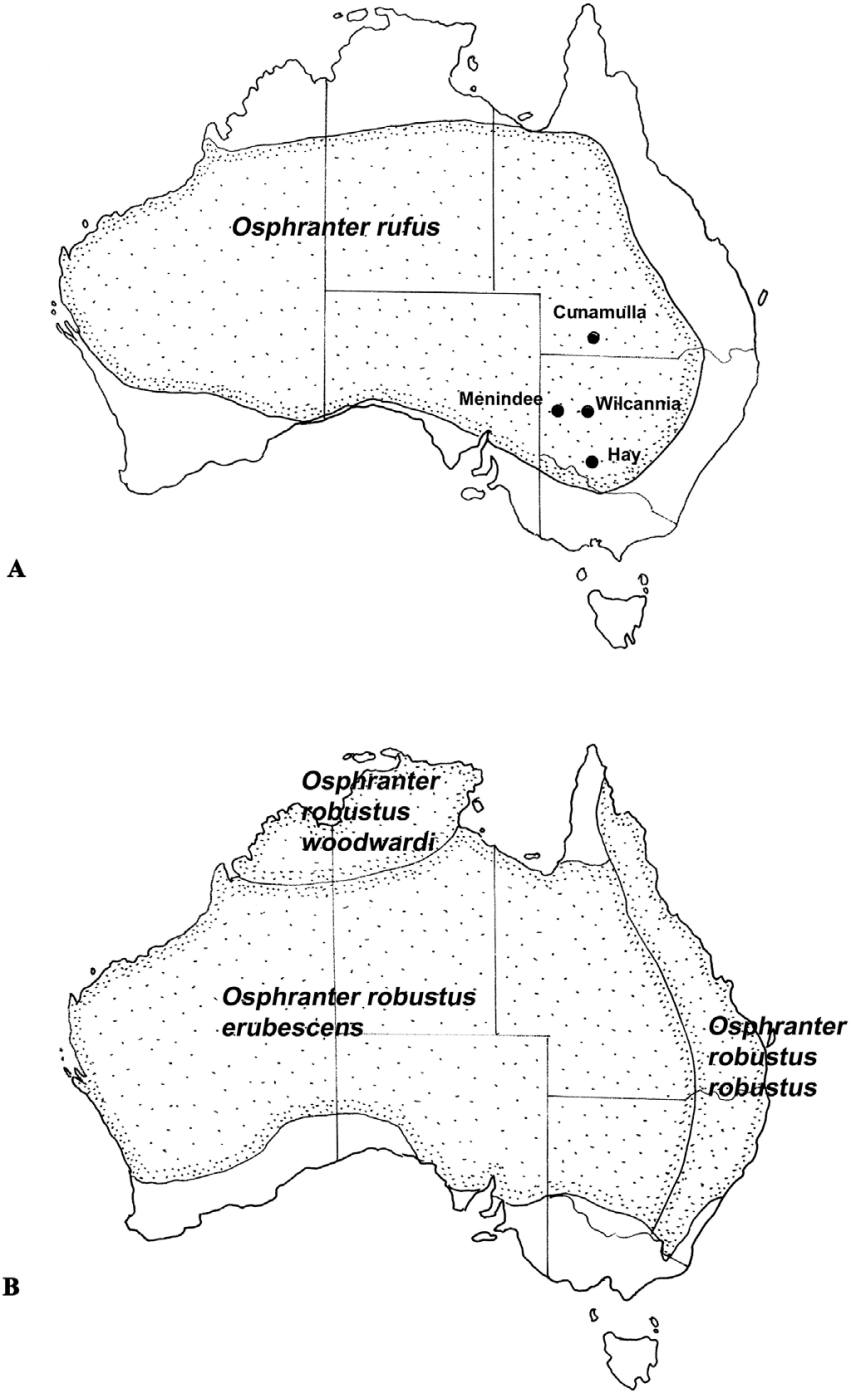
Geographical distributions of the arid adapted kangaroos, the red kangaroo (*Osphranter rufus*) (A) and the euro (*Macropus robustus erubescens*) (B) with its related sub-species, the eastern wallaroo (*M. r. robustus*) and the northern wallaroo (*M. r. woodwardi*), occurring in higher rainfall areas to the east and north of the arid zone respectively. Named localities are sites at which epidemiological studies of the parasites of these kangaroo species have been undertaken.

Adaptation of the parasites of kangaroos and wallabies to the current arid climatic conditions prevailing in central Australia has to be viewed against the evolution of the host groups. The early macropodids were inhabitants of rainforest and these are currently represented by three species of small wallabies colloquially referred to as pademelons (*Thylogale* spp.) which are found in the tropical and sub-tropical rainforests of the east coast of Australia as well as in the temperate forests of Tasmania (van Dyck and Strahan, 2008) (Fig. 2A). Tree kangaroos (*Dendrolagus*) also occur in the tropical rainforests, but these are a more recent evolutionary development (Meredith et al., 2008) and being primarily arboreal present difficulties in comparisons with other terrestrial macropodid species. Common inhabitants of the eastern and southern coastal forests and grasslands are the grey kangaroos *Macropus giganteus* (eastern grey kangaroo) and *M. fuliginosus* (western grey kangaroo). Both species occur not only in the high rainfall coastal zones, but are also common in the intermediate semi-arid zones of Nix (1982) and extend into the arid zone, but only at the limits of their distributions (Fig. 2B). Rainfall patterns (uniform, winter or summer predominance) appear to limit the distributions of these kangaroo species (Caughley et al., 1987). Within the arid zone, the two principal large macropodids are the red kangaroo (*Osphranter rufus*) and the euro (*Ospharanter robustus erubescens*). The distribution of *O. rufus* appears to be limited by mean annual rainfall and mean annual temperature (Caughley et al., 1987) (Fig. 3) but comparable studies are lacking for the euro. The remaining two subspecies of *O. robustus* are *O. r. robustus*, restricted to the higher rainfall regions of the east coast and *O. r. woodwardi* restricted to the monsoonal tropics of northern and western Australia (Fig. 3) (van Dyck and Strahan, 2008).

A review of the helminth parasite faunas of these species allows some comparison between macropodid species inhabiting either coastal rainforests (*Thylogale* spp.), coastal regions of high rainfall and the semi-arid landscapes (*Macropus* spp.) and those occurring primarily in the arid zone (*Osphranter* spp.), although there is considerable overlap in the distributions of latter two genera.

In each host species, the gastrointestinal helminth fauna is dominated by strongylid nematodes, principally located in the complex sacculated forestomach, with a small number of species of strongylid and oxyurid nematodes in the colon and again a small number of species of anoplocephalid or davaineid cestodes in the small intestine and bile ducts (Arundel et al., 1979; Beveridge and Arundel, 1979; Beveridge et al., 1992, 1998; Griffith et al., 2000).

The two species of pademelon (*Thylogale*) occurring on the Australian mainland in rainforest areas along the eastern coast have a highly diverse helminth community with 40 species of nematodes and six species of cestodes (Griffith et al., 2000), none of which are shared with species of *Macropus* and *Osphranter* apart from the metacestode of the introduced cestode, *Echinococcus granulosus*. In the case of *Hypodontus macropi*, a species apparently shared with other macropodids, genetically distinct but morphologically cryptic host specific species are included within this taxon (Jabbar et al., 2013). A notable feature of the communities of gastrointestinal parasites in pademelons is that while a number of nematode genera are shared with other kangaroo species, an additional series of genera in pademelons (*Amphicephaloides*, *Cassunema*, *Foliostoma*, *Tethystrongylus*, *Thallostonema*, *Thylonema*, *Thylostrongylus*, *Trigonostonema*) is restricted to these hosts (Table 2). In addition, only three species of *Cloacina* have been recorded from these hosts (*C. dahli*, *C. cloelia*, *C. cybele*) while in other macropodid hosts species ‘flocks’ of *Cloacina* are generally present (Beveridge et al., 2002) (see below). The anoplocephalid cestodes of pademelons are unique to these hosts (Spratt and Beveridge, 2016) while the davaineid genus *Calostaurus* is restricted to pademelons and in Papua New Guinea to scrub wallabies (*Dorcopsis*) (Spratt and Beveridge, 2016).

**Table 2:** Comparison of nematode genera present in macropodids inhabiting Australian rainforests (*Thylogale*), with those inhabiting sclerophyll forests and grasslands (*Macropus*) and the arid interior (*Osphranter*).

	*Thylogale*	*Macropus*	*Osphranter*
*Alocostoma*	–	+	+
*Amphicephaloides*	+	–	–
*Cassunema*	+	–	–
*Cloacina*	+	+	+
*Coronostrongylus*	(+)^a^	+	+
*Filarinema*	+	+	+
*Foliostoma*	+	–	–
*Globocephaloides*	–	+	–
*Hypodontus*	+	–	+
*Labiomultiplex*	+	–	–
*Labiosimplex*	+	+	+
*Labiostrongylus*	+	–	+
*Macropicola*	–	+	–
*Macroponema*	–	+	+
*Macropostrongyloides*	–	+	+
*Macropostrongylus*	–	–	+
*Monilonema*	+	–	–
*Papillostrongylus*	–	+	+
*Paramacropostrongylus*	–	+	–
*Pharyngostrongylus*	+	+	+
*Popovastrongylus*	+	+	+
*Rugopharynx*	+	+	+
*Sutarostrongylus*	+	–	–
*Tethystrongylus*	+	–	–
*Thallostonema*	+	–	_
*Thylonema*	+	–	–
*Thylostrongylus*	+	–	–
*Trigonostonema*	+	_	_
*Wallabinema*	+	–	–
*Zoniolaimus*	(−)^a^	–	+

a Indicates uncommon occurrence.

The grey kangaroos, *M. fuliginosus* and *M. giganteus*, occupy forests and grasslands in the high rainfall zones, but extend conspicuously into the semi-arid zones fringing the central arid zone and extend to a limited degree into the arid zone itself (Fig. 2B). The gastro-intestinal helminth communities of the grey kangaroos are highly diverse (22 genera, 50 species; 21 genera, 53 species respectively) (Spratt and Beveridge, 2016). Both communities are dominated numerically by gastric inhabiting species of *Rugopharynx* (*R. australis*, *R. macropodis* and *R. rosemariae*) which can be present in very large numbers (13,000 to 294,000 (mean 86,000) in *M. fuliginosus*, and 200 to 268, 000 (mean 50,800) in *M. giganteus*; Beveridge and Arundel, 1979). In addition, each kangaroo species harbours a ‘flock’ of *Cloacina* species, with 15 species recorded from *M. giganteus* and 14 from *M. fuliginosus*, many of which are shared between these two host species (Spratt and Beveridge, 2016). Few of the genera encountered are specific to the grey kangaroos. The helminth communities in the grey kangaroos are therefore significantly different to those found in the rain-forest inhabiting pademelons (Table 2).

Of the two large macropodid species found in the arid zone, the helminth parasites of the red kangaroo, *O. rufus*, are better studied than those of the euro, *O. r. erubescens*. The helminth parasites of *O. rufus* have been examined at several sites (Hay and Wilcannia in New South Wales and Cunnamulla in Queensland) by Mykytowycz (1964) and at Menindee in New South Wales by Arundel et al. (1979) (Fig. 3A). The helminth community of *O. rufus* is diverse (18 genera, 31 species) and in the non-tropical parts of its range is dominated numerically by *R. australis* in the stomach, again frequently in large numbers (9–265,000 (mean 47,500); Arundel et al., 1979), as is the case in the grey kangaroos. This species co-occurs with large numbers of two species of *Zoniolaimus* (250–91,000 (mean 7450); Arundel et al., 1979) and five species of *Cloacina* specific to or primarily parasitic in *O. rufus* (*C. ares*, *C. liebigi*, *C. hydriformis*, *C. eris*, *C. xerophila*).

Studies of the euro are more limited. The helminth records of *O. robustus* were considered at the species level by Spratt and Beveridge (2016) and the only prevalence data available are for *O. r. robustus* (Beveridge et al., 1998). From the limited data available, it appears that the arid zone sub-species, *O. r. erubescens*, harbours 35 helminth parasites with the gastric community dominated by host specific species of *Cloacina* (19 species). *Rugopharynx australis* is also present in 32% of animals, but mainly in the southern part of the host range (Beveridge, 2020). In comparing the helminth fauna of the arid adapted *O .r.* *erubescens* and the nominal subspecies found in higher rainfall areas of the east coast, the similarity of the helminth fauna between the eastern wallaroo, *O. r. robustus,* and the euro, *O. r. erubescens,* (using the reciprocal of Simpson's Index) was 67%, while the similarity between the northern wallaroo, *O. r. woodwardi*, and *O. r. erubescens* was 55% (Beveridge, 2020).

Comparing the helminth communities of these macropodids, it appears that the major difference between rainforest inhabitants and the grazing macropodids is a change in the nematode genera present from one dominated by endemic genera in the rainforest inhabitants, with few species of *Cloacina*, to a pattern in the remaining kangaroos of communities dominated by *Rugopharynx* and *Cloacina*, although there is significant variation between host species in the drier areas. The ecological components of this shift are clear, but the possible involvement of phylogenetic factors is more difficult to determine. Information on the phylogeny of the nematode fauna is extremely limited, but molecular studies to date (Chilton et al., 2018) suggest that the nematode genera unique to pademelons (*Thylogale* spp.) are not monophyletic but rather are scattered through the clades of genera belonging to the Cloacininae and therefore that phylogenetic associations may not be the dominant determination of these distributions. No comparable information is available for the cestodes.

Differences between the forest-grassland-dwelling grey kangaroos and the arid zone red kangaroos and euros appear to be much more subtle and are more likely to be reflected in the species present rather than in the genera. Consequently, these differences are explored in greater detail.

**Fig. 4:**
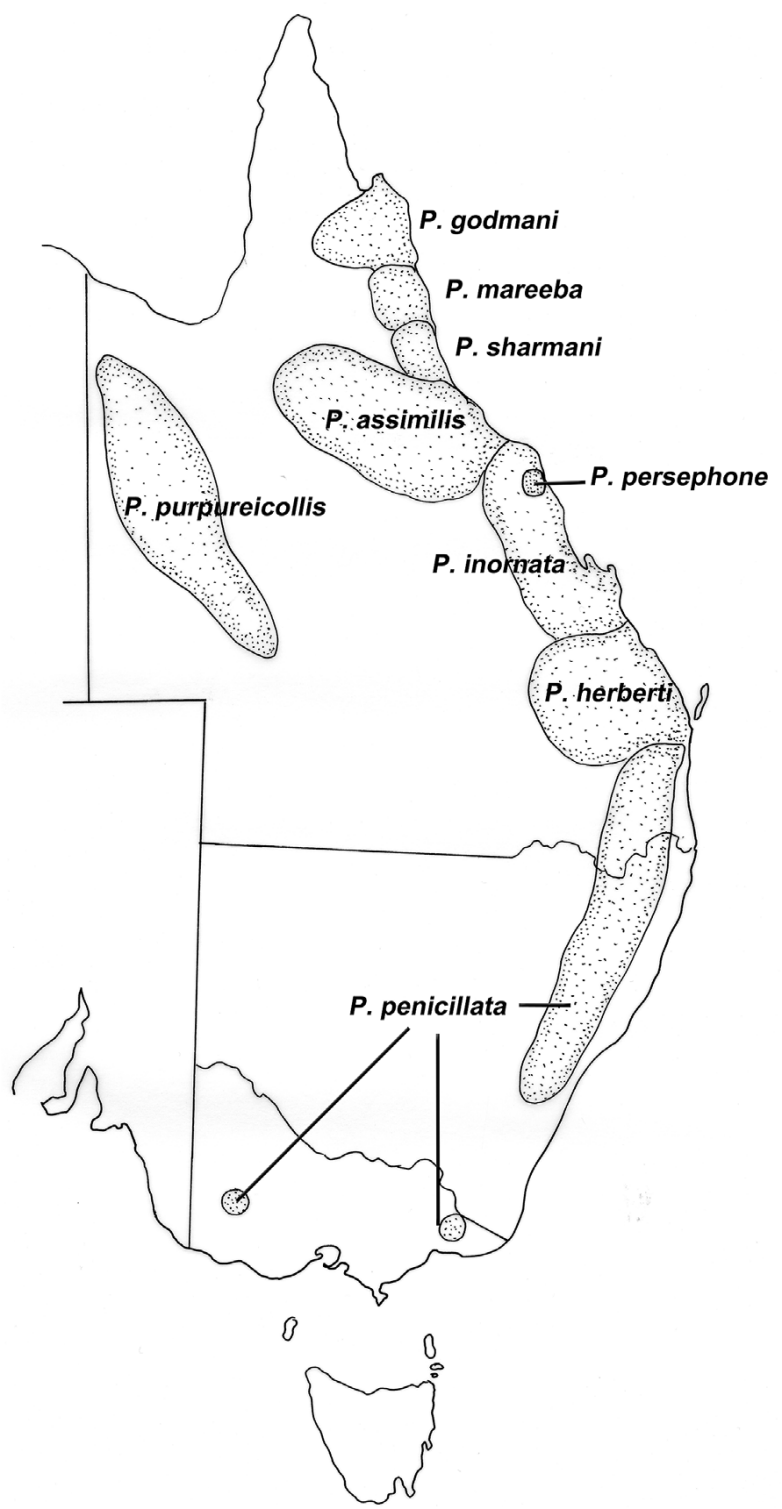
Geographical distributions of species of rock wallaby (*Petrogale*) (Macropodidae) in eastern Australia, comparing that of the rain-forest inhabiting *P. persephone*, with the closely related members of the *P. penicillata* species complex (*P. assimilis*, *P. inornata*, *P. godmani*, *P. mareeba*, *P. penicillata*, *P. sharmani*) occurring in a parapatric pattern along the east coast, and *P. purpureicollis*, the most arid-adapted species.

**Fig. 5:**
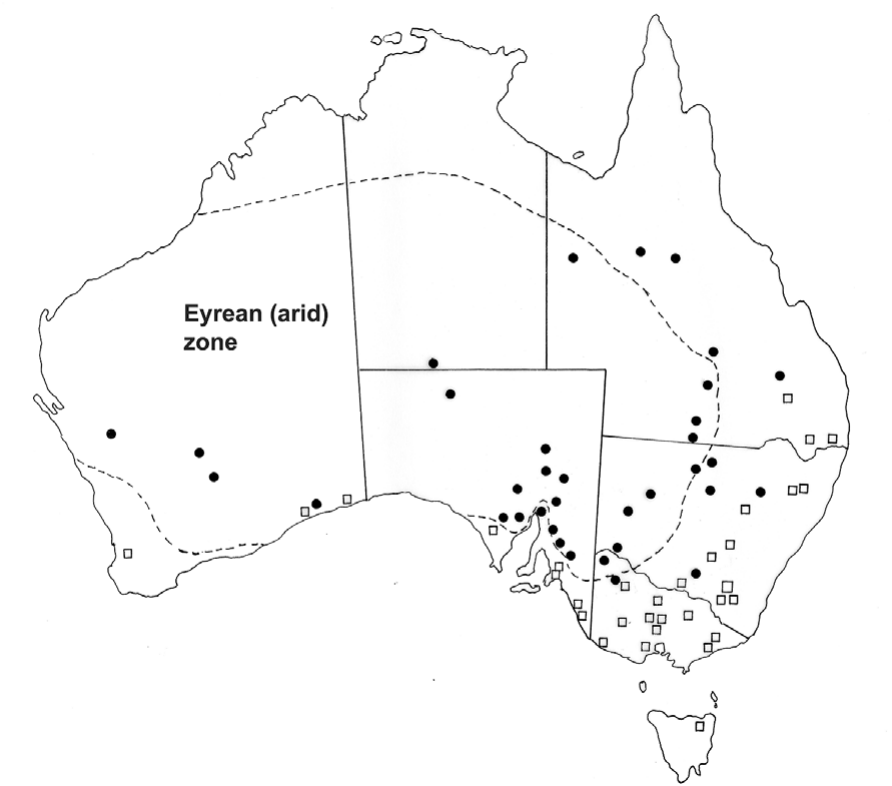
Geographical distributions of the closely related strongylid nematode species *Rugopharynx australis* and *R. macropodis*. Records based on Beveridge and Chilton (1999) and voucher specimens deposited in the South Australian Museum, Adelaide. *Rugopharynx australis* (represented by closed circles) occurs in the stomachs of *Macropus fuliginosus*, *M. giganteus*, *Osphranter rufus* and *O. robustus*; *R. macropodis* (represented by open squares) occurs in the stomachs of *M. fuliginosus* and *M. giganteus*.

*Rugopharynx australis* and the closely related species *R. macropodis* are the most abundant nematodes in both the grey kangaroos and the red kangaroo respectively. Until recently, both species were included within *R. australis* (Beveridge and Chilton, 1999) and *R. australis* is still potentially a composite species (Chilton et al., 2016). The distribution of *R. macropodis* is essentially coastal and montane, while that of *R. australis* is restricted to the interior arid and semi-arid regions of the continent while being found in all four kangaroo species (Beveridge and Chilton, 1999) (Fig. 4). At some localities in the semi-arid zone, both species occur in the same individual kangaroo. That the boundary between the two is indistinct is not surprising given the vagility at least of the red kangaroo (Denny, 1982) and the fact that kangaroo distributions overlap quite significantly. These distributions need to be treated with some caution as there are large expanses of the interior of the continent for which no parasitological data are available. No studies of the biology of these nematode species have been undertaken, but there are relatively well-studied parallels in the species of *Trichostrongylus* found in sheep in these same areas which may provide reasons for such differences in distributions. Of the three species present, *T. colubriformis*, *T. rugatus* and *T. vitrinus*, both Beveridge and Ford (1982) in South Australia and de Chaneet and Dunsmore (1988) in the south west of Western Australia found that *T. rugatus* was much more prevalent in semi arid areas while *T. vitrinus* was the dominant species in areas of high rainfall. *T. colubriformis*, which is dominant in tropical and sub-tropical areas of Australia was present in low numbers only. The differences in distributions have been attributed to the ability of the third stage larva to withstand high temperatures and low relative humidities (Beveridge et al., 1989a). Similar studies on species of *Rugopharyx* may reveal comparable mechanisms determining geographical distributions. A parallel situation occurs in the case of two closely related species of *Cloacina*, *C. hermes* and *C. hestia*, found in the grey kangaroos, with the former occurring in high rainfall areas and the latter in semi-arid areas (Beveridge, 1998) (see Fig. 5).

*Cloacina* is the largest genus of nematodes present in macropodids with 132 species described to date (Beveridge, 2019). Of these, 22 species (17%) occur primarily within the arid zone, with another 12 species (9%) extending into the arid zone. With no robust phylogeny for the genus, it is difficult to draw conclusions about speciation, but it does appear that for this genus, the arid zone has been a significant centre of diversification, whatever the mechanism. The pattern displayed by this genus of nematodes therefore contrasts starkly with the conclusion of Baverstock (1982) that the arid zone has not been a significant area for mammal diversification. The geographical distributions of species of *Cloacina* occurring in *O. robustus* have been examined (Beveridge, 2020) and several obvious patterns identified. Some species of *Cloacina* are found across all climatic zones, others are restricted to the monsoonal tropics of northern Australia and others to the eastern ranges while a small number of species is restricted to the arid zone. These preliminary data suggest that climate may play a significant role in the distribution of the nematode species found in *O. robustus*.

Very little is known about the life cycle of species of parasites found in the arid zone of Australia. In the case of the hookworm -like strongylid nematode, *Hypodontus macropi*, found in the ileum, caecum and colon of red kangaroos, the first stage larva hatches from the egg but when moulting to the second retains the sheath of the first stage larva and the same happens in the second moult such that the infective third stage larva has two sheaths, thus potentially increasing its resistance to dessication (Beveridge, 1979). However, a similar developmental strategy has also been reported in *Labiomultiplex eugenii* in the tammar wallaby (*Notamacropus eugenii*) in higher rainfall areas in South Australia (Smales, 1977) and in *Rugopharynx rosemariae* from grey kangaroos in higher rainfall areas (Beveridge and Presidente, 1978). Consequently, this form of larval development may not be restricted to nematodes in the arid zone. Additional life cycle studies are required to determine how widespread this phenomenon is.

Additional adaptational strategies potentially indicate the way that parasites utilise climatic patterns to ensure transmission. This is best demonstrated in a comparison between the ecological differences between the parasites of red kangaroo at Menindee, New South Wales (in the arid zone) (Arundel et al., 1979) and those of the eastern grey kangaroo in Victoria, in a higher, winter rainfall zone in Victoria (Arundel et al., 1990; Cripps et al., 2015). In Victoria, summers are hot and dry, with maximum rainfall occurring during winter. As a consequence, acquisition of infective larvae from pasture is highly seasonal and occurs during winter and spring (Arundel et al., 1990; Cripps et al., 2015). By contrast, in arid Australia, rainfall is non-seasonal and therefore, no seasonal effects are obvious in terms of parasite abundance/intensity (Arundel et al., 1979) for parasites such as *R. australis*. However, Mykytowycz (1964) did show a highly significant seasonal effect in the case of *Labiosimplex longispicularis* in red kangaroos, and to a lesser extent in the case of *Filarinema flagrifer*. In *L. longispicularis*, larval development within the gastric mucosa was prolonged and occurred over the summer months when external environmental conditions were not conducive for larval development, resulting in gravid females being present only during the winter months when eggs deposited in faeces were more likely to develop to infective larvae and larvae were more likely to persist in the environment, assuming that some rainfall occurred during this period. This may be an adaptation to arid environments. However, a similar highly seasonal strategy of development is also employed by *Labiomultiplex eugenii* in the tammar wallaby, *Notamacropus eugenii* (Smales and Mawson, 1978), which does not occur in an arid environment, and the seasonal development of *Labiosimplex* spp. in grey kangaroos, used here as a comparison, has not been investigated to date.

Rock wallabies (*Petrogale*) as the name implies are wallabies whose primary habitat is rocky outcrops. They occur across the continent often in highly disjuct populations and currently 17 species are recognised (Jackson and Groves, 2015). Of these, four species or races occur in the arid zone: *P. lateralis* (MacDonnell Ranges race), *P. purpureicollis*, *P. rothschildi* and *P. xanthopus* and of these, only *P. purpureicollis* has been examined for parasites in any systematic fashion (15 animals) (Beveridge et al., 1989b; Bradley et al., 2000) while for the remaining species, parasite records are either fragmentary or non-existent. The data available do however allow a comparison between the helminth community of the purple necked rock wallaby, *P. purpureicollis* restricted to the arid zone with that of the members of the *P. penicillata* species complex, *P. assimilis*, *P. godmani*, *P. herberti*, *P. inornata*, *P. mareeba* and *P. sharmani* (see Eldridge et al., 2001; Potter et al., 2012), which occur in the humid zone along the eastern coast of Queensland in a parapatric fashion and for which the helminth communities have been defined (Beveridge et al., 1989b; Griffith et al., 2000; Bradley et al., 2000). The Proserpine rock wallaby, *P. persephone*, is the only rock wallaby species which is a closed forest/rain-forest inhabitant (van Dyck and Strahan, 2008) and is a relict of a once more widely distributed species, but allows a similar comparison of helminth faunas as was undertaken for the other macropodids. The helminth communities of rock wallabies are similar to those found in the larger kangaroos and differ only at the specific level, with a similar range of genera present. The number of species of helminths present in members of the *P. penicillata* complex (represented by the examination of 10 or more individuals) ranges from 10 to 21 with gastric nematode burdens ranging from 3100–12,450 (Beveridge et al., 1989b), with comparable values from the rain-forest inhabiting *P. persephone* being 19 species and a mean gastric nematode burden of 23,300 respectively (Griffith et al., 2000). In *P. purureicollis*, from the arid zone, 13 helminth taxa are present and average number of gastric nematodes is 6150 (Bradley et al., 2000). Therefore, there are roughly similar numbers of helminth taxa and intensities of infection in the arid zone representative. The helminths of the individual species of the *P. penicillata* complex of rock wallabies show a remarkable degree of similarity (Beveridge et al., 1989b), with communities in *P. assimilis*, *P. godmani*, *P. herberti* and *P. inornata* having overall similarities of 55% or higher (Beveridge et al., 2010). Species of this complex shared approximately 35% of their helminth species with *P. persephone* and only 25% with *P. purpureicollis* (Beveridge et al., 2010). These overall similarity estimates include all helminth species, not only those specific to rock wallabies. The nematode species specific to rock wallabies or that occur primarily in rock wallabies but are found only occasionally in sympatric hosts are shown in Table 3. There are genera and species restricted to either the closed forest/rain-forest inhabiting *P. persephone*, members of the *P. penicillata* complex and to *P. purpreicollis* in the arid zone. Two of the nematode genera present in *P. persephone* do not occur in the other rock wallabies, while an additional seven species found in *P. persephone* (Spratt and Beveridge, 2016) are not included in the table as they are shared with the pademelon, *Thylogale stigmatica*, with which *P. persephone* shares its habitat. The extent to which this is an ecological phenomenon or a phylogenetic one, since *P. persephone* is potentially phylogenetically close to the pademelons, is not clear (Griffith et al., 2000).

**Table 3:** Nematode parasites of rock wallabies inhabiting rain-forest (*Petrogale persephone*), humid coastal environments (*Petrogale penicillata* complex) and the arid zone (*P. purpureicollis*).

	*P. persephone*	*P. penicillata complex*	*P. purpureicollis*
Only in *P. persephone*			
*Hypodontus macropi*^a^	+	–	–
*Thallostonema queenlandense*	+	–	–
*Coronostrongylus closei*	+	–	–

Only in *P. penicillata* complex			
*Cloacina idas*	–	+	–
*Cloacina petronius*	–	+	–
*Coronostrongylus sharmani*	–	+	–
*Coronostrongylus spratti*	–	+	–
*Labiosimplex godmani*	–	+	–
*Labiosimplex petrogale*	–	+	–
*Macropostrongylus petrogale*	–	+	–
*Rugopharynx petrogale*	–	+	–
*Rugopharynx zeta*	–	+	–
*Sutarostrongylus petrogale*	–	+	–

Only in *P. purpureicollis*			
*Cloacina ernabella*	–	–	+
*Cloacina petrogale*	–	–	+
*Rugopharynx alpha*	–	–	+

In *P. persephone*/*P.penicillata*			
*Sutarostrongylus safestatus*	+	+	–
*Zoniolaimus petrogale*	+	+	–

In *P. penicillata*/*P. purpureicollis*			
*Cloacina caenis*	–	+	+
*Cloacina pearsoni*	–	+	+

*In all groups*			
*Cloacina robertsi*	+	+	+

a Distinct genetic form.

Some of these nematode species, particularly those found in members of the *P. penicillata* complex are shared with *P. purpureicollis* in the arid zone (although recent genetic studies of some of the species of *Cloacina* suggest that *C. caenis*, *C. pearsoni* *and* *C. robertsi* are likely to represent sibling species complexes (Chilton et al., 2009)). However, additional species (eg. *C. elegans*, *C. ernabella, C. petrogale*) as well as *Rugopharynx alpha* are found only in rock wallabies inhabiting the arid zone (*P. purpureicollis*, *P. lateralis* MacDonnell Ranges race) (Bradley et al., 2000; Beveridge, 2019) suggesting some shift in the fauna towards arid adapted species.

The overall conclusion that can be drawn from an analysis of the helminth communities in representatives of the macropodids is that while there are differences in the composition of communities between rain-forest dwellers and non-rain-forest dwellers, the differences between those inhabiting forests and open woodlands in the humid zone but extending into the semi-arid regions generally differ only in their species composition from those macropodids occurring almost exclusively in the arid zone. It is possible to highlight species pairs such as *R. australis*-*R. macropodis* in which distributions between arid and humid zones are evident, but for most helminth species adequate distribution data are not available. In addition, particularly in the case of the large kangaroos, distributions overlap at the margins of their ranges and the lie within a gradation of climate characters from humid coastal regions to drier inland regions with particular overlap in semi-arid regions.

Among ectoparasites, kangaroos and wallabies are hosts to a number of species of ixodid ticks in the coastal humid zones of Australia (Roberts, 1970). However, only a single subspecies of the kangaroo tick, *Amblyomma triguttatum*, *A. t. rosei* extends into the arid zone (Roberts, 1962). The remaining sub-species of this tick are found in coastal humid zones (Roberts, 1962). Most of the current collection records of this sub-species are from cattle, but as this species is primarily a parasite of kangaroos, it is assumed that the normal host will be the red kangaroo, *O. rufus*, with cattle as secondary hosts. Guglielmone (1994) and Waudby and Petit (2007) studied the biology of the nominate sub-species on the east coast of Australia and in South Australia, but there are no currently available comparable data for *A. t. rosei* to indicate how it might have adapted to conditions prevailing in the arid zone.

The argasid or ‘soft tick’ *Ornithodoros gurneyi* is a common tick parasitising kangaroos exclusively within the arid zone (Browning, 1962). It is found in the soil or sand of areas where kangaroos (mainly the red kangaroo, *O. rufus*) “camp” during the day under isolated trees on open plains. It feeds rapidly on resting kangaroos, returning to its subterranean environment to moult or breed (Doube, 1975a). Two forms of this species exist, one found in the “camp sites” of *O. rufus* and the other in the caves inhabited by the wallaroo, *O. robustus* (Doube, 1975b). This tick, as is the case with a number of its congeners (e.g. *O. moubata*, the ‘tampan tick’ of Africa) is ideally suited to surviving in arid environments.

Thus, while the differences between the internal helminth parasites of kangaroos inhabiting coastal/semi-arid environments are relatively subtle, there are more potentially more striking differences in their ectoparasites.

## 6 The wombats (Vombatidae)

**Fig. 6:**
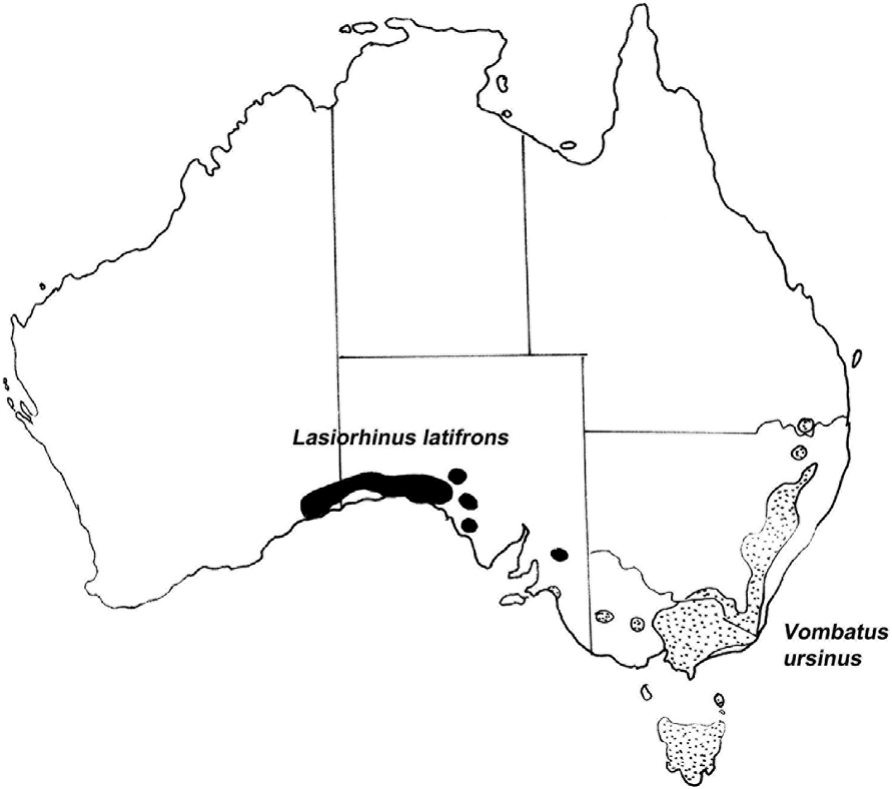
Geographical distributions of two species of wombats (Marsupialia: Vombatidae). The stippled area represents the distribution of the common wombat, *Vombatus ursinus*, in forested areas of south-eastern Australia; the areas in black indicate the distribution of the southern hairy-nosed wombat, *Lasiorhinus latifrons*, in the semi-arid and arid areas of South Australia.

There are three extant species of wombat, the common or smooth-nosed wombat, *Vombatus ursinus*, occurring in high rainfall areas of south-eastern Australia including Tasmania (van Dyck and Strahan, 2008), the southern hairy nosed wombat, *Lasiorhinus latifrons*, found in arid and semiarid parts of South Australia (van Dyck and Strahan, 2008) (Fig. 6) and the endangered northern hairy-nosed wombat, *L. krefftii*, restricted to an isolated area of central Queensland (van Dyck and Strahan, 2008).

Vombatiform marsupials were common in the ancient rainforests of central Australia (Hope, 1982) with the extant species relicts of a much more significant evolutionary radiation.

Data of helminth communities are available for only two of the extant species, but they allow a comparison between forest-adapted and arid adapted host species. The dominant gastro-intestinal helminths of wombats are the strongylid nematodes of the genera *Macropostrongyloides*, *Phascolostrongylus* and *Oesophagosomoides* which occur in the colon. Quantitative data are limited, but burdens of 1000–6000 *O. stirtoni* and 200 *M. lasiorhini* have been reported in *L. latifrons* (Beveridge, 1978). Skerratt (1998) reported up to 2250 *Phascolostrongylus*, 900 Oes*. longispicularis* and 400 Oes*. giltneri* in *V. ursinus* from the Healesville region of Victoria. Beveridge (1978) reported about 6600 nematodes in a single wombat comprising 20% *Phascolostrongylus* and 80% *Oesophagostomoides*. Apart from *Phascolostrongylus* which is restricted to *V. ursinus* (Beveridge, 1978), the remaining genera are shared, although with different species of nematodes in the two host species (Beveridge, 1978; Beveridge and Mawson, 1978; Sukee et al., 2019). The two species of wombats share the anoplocephalid cestode *Phascolocestus johnstoni* in the small intestine, with *Phascolotaenia comani* restricted to *V. ursinus* and two species of *Progamotaenia* (*P. diaphana* and *P. vombati*) occurring in the bile ducts of the two species of wombats. Thus, the gastrointestinal helminth fauna of the two species of wombats is quite similar showing relatively few differences between humid and semi-arid environments. However, life cycle studies would be necessary to elucidate the ways in which species of nematodes have adapted to the semi-arid environments inhabited by *L. latifrons*.

As with the kangaroos, there are striking differences in the ectoparasite fauna of the two genera of wombats. Apart from the introduced mite *Sarcoptes scabiei*, which is found in both wombat species (Martin et al., 1998; Ruykys et al., 2009), the most common and prominent ectoparasite of *V. ursinus* is the tick *Bothriocroton auruginans*. Specific to wombats, Skerratt (1998) reported a high prevalence of this tick in the Healesville region of Victoria with 55% of wombats examined having more than 50 ticks. By contrast, this tick is not found/rare on *L. latifrons* (Roberts (1970) includes it as a host, but provides no collection data). Several species of *Ixodes*, typical of species occurring in humid coastal zones, occur on *V. ursinus*, but are not present on *L. latifrons* (Roberts, 1970).

By contrast, *L. latifrons* is infested with five species of stick-fast fleas of the genus *Echidnophaga*, (*E. calabyi*, *E. cornuta*, *E. eyrei*, *E. octotricha*, *E. perilis*) three of which (*E. calabyi*, *E. cornuta*, *E. octotricha*) are only found on this species of wombat. *Echidnophaga eyrei* is also found on *V. ursinus* and *E. perilis* is found on a variety of host species (Dunnett and Mardon, 1974). Of the 20 known species of *Echidnophaga,* 10 are endemic to Australia and occur primarily in arid and semiarid regions (Dunnett and Mardon, 1974), suggesting that this genus may be specifically adapted to arid conditions.

The wombats, as with the large macropodids, show only minor differences between forest inhabiting and semi-arid inhabiting species in terms of their internal parasites, the differences generally being at the specific level. Unfortunately, the wombat precursors inhabiting the former central Australian rainforests are extinct and consequently nothing is known of their helminth parasite fauna. By contrast, as with the macropodids, their ectoparasite fauna appears to be distinctive, with ticks the dominant parasites of the common wombat, while the arid-adapted genus of fleas, *Echidnophaga*, appears to be the most common ectoparasite group found on southern hairy nosed wombats.

## 7 Marsupial moles

Two species of marsupial mole are currently recognised, *Notoryctes typhlops* and *N. caurinus* (Jackson and Groves, 2015). Both live in sand dunes in central Australia and little is known of their biology (van Dyck and Strahan, 2008). Only a few moles (*N. typhlops*) have ever been examined for helminth parasites and the genera of trichostrongyloid nematodes found in them, *Austrostrongylus* and *Nicollina* (Beveridge and Durette-Desset, 1985), have congeners in macropodids and echidnas respectively (Spratt and Beveridge, 2016) an enigmatic association which currently remains to be explained. However, most trichostrongyloid nematodes have free-living stages (Anderson, 2000) and therefore how the species found in marsupial moles manage to complete their life cycles within tunnels in sand dunes in central Australia also remains an enigma.

## 8 Rodents

The Australian rodent fauna is the result of multiple invasions from Papua New Guinea within approximately the last five million years (Rowe et al., 2008, p. 96). The so-called ‘old endemic” genera of rodents are placed in three tribes, Conilurini, Hydromyini and Uromyini. The ‘new endemics”, comprising the genus *Rattus* arrived possibly one million years ago. The Hydromyini contains the ‘water rats’, *Hydromys* and *Xeromys* while the Uromyini contains the so-called ‘mosaic-tailed rats’ including the genera *Melomys* and *Uromys*, species which are primarily arboreal and found most commonly in coastal forests including rainforests (van Dyck and Strahan, 2008). The related tribe Conilurini contains a number of genera among which are two large genera, *Pseudomys* and *Notomys* containing many species found in highly arid areas as well as a small genus *Zyzomys*, known as rock rats, which occur in rocky outcrops across northern Australia (see Fig. 6).

**Fig. 7:**
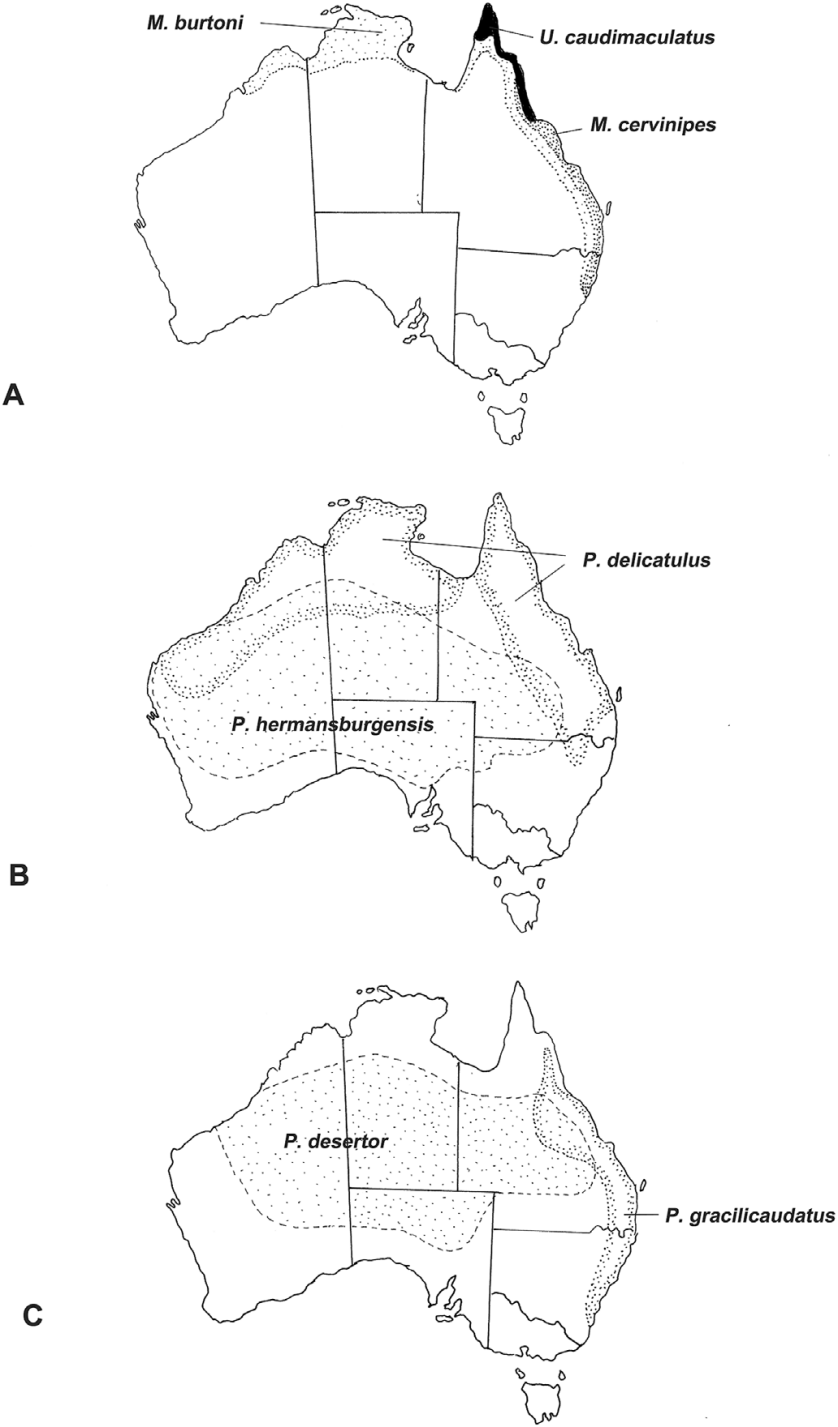
Distributions of selected endemic species of hydromyine rodents in Australia for which published helminthological studies are available. A, Distributions of the grassland melomys, *Melomys burtoni*, the fawn-footed melomys, *M. cervinipes* and the white-tailed rat, *Uromys caudimaculatus*; B, Distributions of the delicate mouse, *Pseudomys delicatulus* and the sandy inland mouse, *P. hermansburgensis*; C, Distributions of the eastern chestnut mouse, *P. gracilicaudatus* and the desert mouse, *P. desertor*.

The helminth fauna of Australian rodents remains relatively poorly studied (Smales, 1997), particularly species inhabiting the arid zone, but studies of the helminth communities for the forest dwelling genera, *Melomys* and *Uromys* (Smales, 2005; Smales et al., 2004; Smales and Spratt, 2008) allow comparison with some species of *Pseudomys* from coastal forests and from the arid zone (Weaver and Smales, 2012). While *Uromys caudimaculatus* is primarily a rain forest inhabitant, *Melomys cervinipes* is found in both rainforests and eucalypt forests along the eastern coast of Australia (van Dyck and Strahan, 2008). The range of *Melomys burtoni*, the so-called “grassland melomys”, extends beyond forests and into grasslands as the vernacular name implies. Of the species of *Pseudomys* which have been studied helminthologically, *P. gracilicaudatus* and *P. delicatulus* are found in higher rainfall areas of the north and east of the continent, while *P. desertor* and *P. hermansburgensis* are primarily inhabitants of the arid zone (Fig. 7). Distributions of the species do however overlap (van Dyck and Strahan, 2008). The data presented in Table 4 suggest a clear differentiation between the helminth communities of genera inhabiting rain-forest genera (*Melomys*, *Uromys*) and the forest/arid zone -inhabiting genus (*Pseudomys*) both in terms of numbers of families and numbers of species encountered. Currently, it is not possible to distinguish between the ecological and phylogenetic factors potentially responsible for these differences, but the helminth communities in *Pseudomys* are quite depauparate when compared with the rain-forest inhabiting genera (Weaver and Smales, 2012). The most striking differences are in the number of heligmonellid species present with their complete absence in the arid zone. The diversity of helminths in *P. hermansburgensis* in the arid zone is due solely to a diversification in the genus *Syphacia.* The heligmonellids have free-living larval stages potentially susceptible to the effects of dessication while in the oxyurids (*Syphacia*) the larva hatches from the ingested highly resistant egg (Anderson, 2000) suggesting that the oxyurids are inherently better suited to transmission in arid environments. The helminth community of *Zyzomys argurus* is similarly depauparate, with dominance by species of *Syphacia* (Weaver and Smales, 2009) but studies on congeners are lacking.

**Table 4:** Helminth communities of rodents from rain forests (*Melomys* spp., *Uromys*), coastal open forest and woodland (*Pseudomys delicatulus*, *P. gracilicaudatus*) and inland Australia (*Pseudomys desertor*, *P. hermansburgensis*). Numerals indicate the number of parasite taxa present.Exotic parasites excluded Identified to genus.

	*M. cervinipes*	*M. burtoni*	*U. caudimaculatus*	*P. delicatulus*	*P. gracilicaudatus*	*P. desertor*	*P. hermansburgensis*
Parasite group							
Cestoda	2	1	3	0	0	0	1

**Digenea**	1	0	0	0	0	0	0
**Nematoda**							
STRONGYLIDA							
Angiostrongylidae	1	1	1	0	0	0	0
Dromaeostrongylidae	2	1	0	0	0	0	0
Heligmonellidae	11	8	11	0	3	0	0
Molineidae	2	1	0	0	0	0	0
OXYURIDA							
Oxyuridae	1	1	0	2	1	1	6
ASCARIDIDA							
Ascarididae (larval)	3	1	1	0	0	0	0
Heterakidae	1	0	0	0	0	0	0
Heteroxynematidae	0	0	0	0	0	0	1
Seuratidae	0	0	1	0	0	0	0
Subuluridae	1	0	1	0	0	0	0
SPIRURIDA							
Onchocercidae	1	0	2	0	0	0	0
Pneumospiruridae	0	0	1	0	0	0	0
Physalopteridae	1	1	0	0	0	0	0
Rictulariidae	0	0	1	0	0	0	0
Spirocercidae	1	1	2	0	0	0	0
Spiruridae	0	0	1	0	0	0	0
RHABDITIDA							
Strongyloididae	1	1	1	0	0	0	0
ENOPLIDA							
Capillariidae	2	1	2	0	0	0	0
Trichuridae	1	1	1	0	0	0	0
ACANTHOCEPHALA							
Plagiorhynchidae	0	0	1	0	0	0	0
Total	31	19	30	2	4	1	8

More general comments on the adaptation of rodent parasites to the Australian arid zone are limited by the almost total lack of studies of the species of *Notomys* and in the case of *Rattus*, by the lack of studies of the sole arid-adapted species *R. villosissimus*.

Conclusions from studies on rodents need to be approached with caution due to the obvious deficiencies of comprehensive studies, but there are some parallels with the studies of macropodids in that there appears to be a significance difference in helminth faunas between those of rain-forest inhabitants and other rodents for which studies are available, with a more subtle difference between helminth communities of rodents in coastal open forests and those in the arid zone. More extensive studies are clearly needed to clarify these differences.

## 9 Conclusions

While the arid-adapted mammalian fauna of Australia clearly harbours extensive communities of both ectoparasites and helminths, the relative lack of documentation of the parasite fauna in the arid zone and the almost total lack of life history and biological studies of this fauna limits the observations that can be made concerning parasite adaptation. However, there are a number of relatively clear patterns. In both macropodids and rodents, the fauna present in rain-forest inhabiting host species is quite different from that found related genera and species inhabiting the open forests or shrublands of the coastal zones and the arid interior. The differences between helminth communities of macropodids, wombats and rodents in coastal, open forests or shrublands and those of the arid zone are less obvious and there are often differences at the parasite species level. Given the ecological history of the Australian continent with the gradual disappearance of rainforests and the increasing aridity in central Australia, these changes are probably not surprising and concord with conclusions from botanical studies which indicate that the flora of the central arid zone is recent in origin and is derived primarily from the surrounding semi-arid areas (Barlow, 1981).

Currently it is possible to identify differences in the helminth and arthropod fauna between the various climatic regions in and around the central Australian arid zone but identifying the biological adaptations of the parasites surviving in these environments must await studies of their biology which are currently almost entirely lacking.

## Declaration of competing interest

The authors declare that they have no known competing financial interests or personal relationships that could have appeared to influence the work reported in this paper.
